# U turn to venous air embolism

**DOI:** 10.4103/0972-5229.56055

**Published:** 2009

**Authors:** Harsimran Singh, Anurag Tewari, Balvinder Kaur, Suchita Garg

**Affiliations:** **From:**1,2,4 Department of Anaesthesiology, Dayanand Medical College and Hospital, Ludhiana, Punjab, India; 3 Department of Paediatric, Ludhiana Medicity Hospital, Ludhiana, Punjab, India

**Keywords:** Glass bottles, IV fluid therapy, venous air embolism

## Abstract

There is a definitive risk of venous air embolism when the fluid infusion is complete and the drip set is still open in a glass bottle. We have devised a novel way of preventing the chances of air embolism when the fluid in the glass bottle finishes. It really gives a “U” turn to the chances of venous air embolism.

## Introduction

Air embolism can complicate peripheral IV fluid therapy[1] or central venous catheter monitoring, including problems with IV infusion pumps,[2] improper flushing of IV sets,[3] incorrect injection of drugs into the infusion system,[3] and accidental disconnection of the hub or removal of central venous catheters.[4] The open to air system in glass bottles is also a potential hazard for life-threatening complications.

Air embolism can cause blockage of small vessels in the pulmonary vasculature with compromise of gas exchange, cessation of ventricular pumping caused by blockage of air and arrhythmia,[5] and paradoxical air embolism to the systemic circulation through a probe-patent foramen ovale.[6]

**Figure 1:**
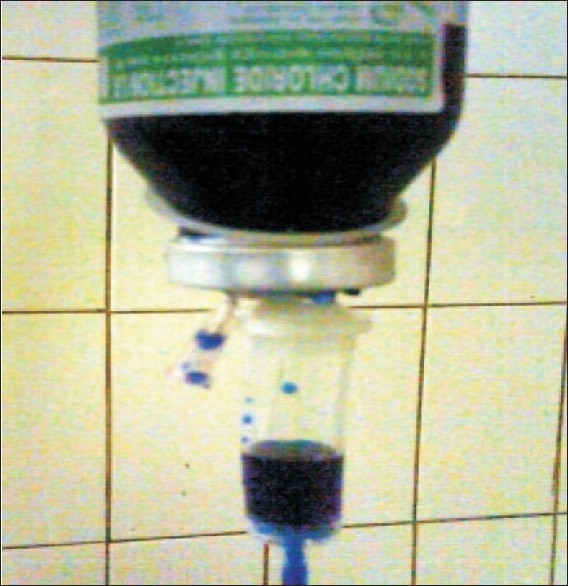
The needle acting as an airway in glass bottle

Glass bottles are widely used for administration of intravenous fluids. Various drugs e.g., mannitol are also available only in glass bottles in our part of the world. For the administration of fluids from glass bottles, a needle has to be inserted, which acts as an airway [Figure 1] and the fluid comes out through the infusion tubing on the basis of the beer can principle. The rate is controlled by a plastic regulating clamp.

Many times the fluid in the bottle finishes, but the regulating clamp is still open. The needle airway is in place and the infusion line eventually becomes open to air, which can lead to fatal air embolism.

For air embolism to occur through an intravenous drip set, there are two prerequisites. First, is that the patient is connected to an intravenous line that is open to air and second is that the intravenous tubing should be placed into a vein whose pressure is below the atmospheric pressure.

Sub atmospheric pressure can develop in a vein when the intravenous tubing is attached to the central line, or the vein is on the upper arm in the lateral position.[7] A right-sided central line in the left lateral position and the patient attempting to breathe against a closed glottis are two other potential conditions of air embolism; there are high chances of sub atmospheric pressure developing in the vein.

There is a definitive risk of air embolism when the fluid infusion is complete and the drip set is still open.

**Figure 2:**
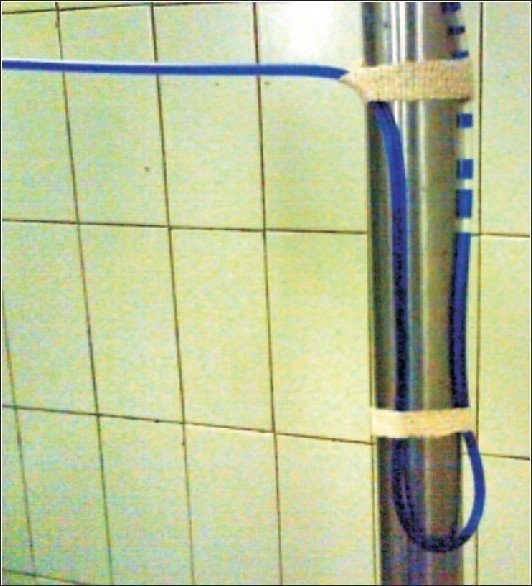
The “U” turn of the infusion set to decrease the chances of an air embolism Methylene dye was put in the IV set for better pictorial presentation

We have devised a novel way of preventing the chances of air embolism when the fluid in the glass bottle finishes. Weix the infusion tubing by giving it a “U” turn so that the loop is below the level of the vein into which the infusion set is attached [Figure 2]. This ensures that some fluid remains in the loop and an air embolism is prevented. The presence of a U turn loop increases the negative pressure that would be required to aspirate air. A simple maneuver acts as a safety mechanism from a potentially life-threatening complication and decreases the chances of an air embolism.

## References

[1] Groell R, Schaffler GJ, Rienmueller R (1997). The peripheral intravenous cannula: a cause of venous air embolism. Am J Med Sci.

[2] Rothenberg F, Schumacher JR, Rosenthal RL (1994). Near-fatal pulmonary air embolus from presumed inadvertent pressure placed on a partially empty plastic intravenous infusion bag. Am J Cardiol.

[3] Levy I, Mosseri R, Garty B (1996). Peripheral intravenous infusion: Another cause of air embolism. Acta Paediatr.

[4] Ross SM, Freedman PS, Farman JV (1979). Air embolism after accidental removal of intravenous catheter. Br Med J.

[5] Adornato DC, Gildenberg PL, Ferrario CM, Smart J, Frost EA (1978). Pathophysiology of intravenous air embolism in dogs. Anesthesiology.

[6] Colohan AR, Perkins NA, Bedford RF, Jane JA (1985). Intravenous fluid loading as prophylaxis for paradoxical air embolism. J Neurosurg.

[7] Plumer's Principles and Practice of Intravenous Therapy (2006). Sharon Weinstein, Ada Lawrence Plumer.

